# CEEMDAN-LWT De-Noising Method for Pipe-Jacking Inertial Guidance System Based on Fiber Optic Gyroscope

**DOI:** 10.3390/s24041097

**Published:** 2024-02-07

**Authors:** Yutong Zu, Lu Wang, Yuanbiao Hu, Gansheng Yang

**Affiliations:** 1 School of Engineering and Technology, China University of Geosciences, Beijing 100083, China; 3002220025@email.cugb.edu.cn (Y.Z.); hyb@cugb.edu.cn (Y.H.); ygansheng@cugb.edu.cn (G.Y.); 2 Technology Innovation Center for Directional Drilling Engineering, Ministry of Natural Resources, Langfang 065099, China; 3 Innovation Base for Directional Drilling Engineering, Geological Society of China, Langfang 065099, China

**Keywords:** pipe-jacking, FOG, CEEMDAN, LWT, noise reduction

## Abstract

An inertial guidance system based on a fiber optic gyroscope (FOG) is an effective way to guide long-distance curved pipe jacking. However, environmental disturbances such as vibration, electromagnetism, and temperature will cause the FOG signal to generate significant random noise. The random noise will overwhelm the effective signal. Therefore, it is necessary to eliminate the random noise. This study proposes a hybrid de-noising method, namely complete ensemble empirical mode decomposition with adaptive noise (CEEMDAN)—lifting wavelet transform (LWT). Firstly, the FOG signal is extracted using a sliding window and decomposed by CEEMDAN to obtain the intrinsic modal function (IMF) with *N* different scales and one residual. Subsequently, the effective IMF components are selected according to the correlation coefficient between the IMF components and the FOG signal. Due to the low resolution of the CEEMDAN method for high-frequency components, the selected high-frequency IMF components are decomposed with lifting wavelet transform to increase the resolution of the signal. The detailed signals of the LWT decomposition are de-noised using the soft threshold de-noising method, and then the signal is reconstructed. Finally, pipe-jacking dynamic and environmental interference experiments were conducted to verify the effectiveness of the CEEMDAN-LWT de-noising method. The de-noising effect of the proposed method was evaluated by SNR, RMSE, and Deviation and compared with the CEEMDAN and LWT de-noising methods. The results show that the CEEMDAN-LWT de-noising method has the best de-noising effect with good adaptivity and high accuracy. The navigation results of the pipe-jacking attitude before and after de-noising were compared and analyzed in the environmental interference experiment. The results show that the absolute error of the pipe-jacking pitch, roll, and heading angles is reduced by 39.86%, 59.45%, and 14.29% after de-noising. The maximum relative error of the pitch angle is improved from −0.74% to −0.44%, the roll angle is improved from 2.07% to 0.79%, and the heading angle is improved from −0.07% to −0.06%. Therefore, the CEEMDAN-LWT method can effectively suppress the random errors of the FOG signal caused by the environment and improve the measurement accuracy of the pipe-jacking attitude.

## 1. Introduction

Long-distance curved pipe-jacking has significant engineering and economic benefits in tunneling projects crossing densely built-up urban areas, heavily trafficked road sections, and oversized cross-sections [1,2]. However, the traditional pipe-jacking guidance method relies on the visibility conditions of the environment and does not apply to long-distance curved pipe-jacking. The fiber optic gyroscope (FOG) is an all-solid-state angular velocity-sensitive device, and it can be sensitive to the angular velocity of the Earth’s rotation, which has the advantage of autonomous navigation [3]. Therefore, the pipe-jacking inertial guidance system uses FOG as a primary sensor to provide real-time attitude measurements for long-distance curved pipe-jacking [4]. But in practical applications, large motor and transformer operations will produce vibrations, electromagnetic disturbances, and other environmental interference due to the rotation of the pipe-jacking cutter. The environment will affect components such as the fiber optic ring in the FOG, and the FOG signal will contain a large amount of random noise, resulting in insufficient long-term accuracy [5]. Therefore, it is necessary to reduce noise to obtain more accurate FOG signals and improve the accuracy of the pipe-jacking attitude [6].

Commonly used FOG random noise filtering methods include digital low-pass filters and time series forecasting methods [7,8]. The time series forecasting method is based on Kalman filtering by establishing an autoregressive (AR) or autoregressive moving average (ARMA) model for the FOG drifting signal, and optimal estimation is performed by a strong tracking Kalman filtering method [9,10]. The Kalman filter cannot strictly distinguish between the useful signal and interference noise in the high frequency part. Therefore, if the low-pass filter is too narrow, it will cause a loss of useful signal, and if the low-pass filter is too wide, it will lead to a poor filtering effect. However, the Kalman filtering method based on AR and ARMA models is proposed for smooth signals. Non-smooth FOG signals need to be smoothed before using these filtering methods.

The empirical mode decomposition (EMD) proposed by Huang E. et al. has achieved effective results in non-smooth and non-linear signal processing [11]. The EMD can adaptively decompose a signal into a series of intrinsic mode functions (IMFs) according to its own characteristics. The IMF is a description of the signal in different scales. Compared with wavelet transform, EMD has desirable adaptivity and decomposition effects in signal processing for non-linear and non-smooth signals. However, the EMD method has an issue that the IMFs may interact or alias with each other during the decomposition process, i.e., the signals of different scales and frequencies appear in the same IMF component, or signals of the same scale and frequency are decomposed into multiple IMF components [12]. Wu et al. [13] proposed an ensemble empirical mode decomposition (EEMD) method to solve the modal aliasing problem. The EEMD method is to decompose the original signal by adding white noise several times, and then averaging the results of the multiple decompositions to obtain the final IMF. Since the EEMD method adds white noise to the original signal several times, it will cause reconstruction errors. Yeh et al. [14] proposed a complementary ensemble empirical mode decomposition (CEEMD) method based on EEMD. The CEEMD works by adding two opposite white noise signals to the original signal for EMD decomposition. The residual white noise in the reconstructed signal is effectively eliminated under the premise of guaranteeing the decomposition effect is comparable to EEMD. However, not only do both EEMD and CEEMD have a problem with computation and storage complexity, but also the decomposition is too dependent on the amplitude of the added white noise and the number of accumulated averages. Thus, Torres et al. [15] proposed complete ensemble empirical mode decomposition with adaptive noise (CEEMDAN), which obtains the IMF by calculating the residual signal by adding adaptive white noise at each stage of the EMD decomposition. The CEEMDAN method has complete decomposition, and the reconstruction error is very small each time, which solves the problem of the low computational efficiency of EEMD and CEEMD, and has a strong adaptability to non-smooth and non-linear signals [16]. Therefore, for the non-linear and non-smooth signals of the FOG in the pipe-jacking inertial guidance system, the CEEMDAN method is suitable for the noise reduction. However, the traditional CEEMDAN method directly removes the high-frequency IMF components for noise reduction, which can easily cause the loss of useful high-frequency signals, and random noise and spurious IMF components may also be contained in the mid-frequency and low-frequency IMF components.

Zhao et al. [17] propose a dynamic error compensation method for FOG based on CEEMDAN that properly separates the effective signal and dynamic error. There is no further algorithm that is added to filter out the noise signal. Wang et al. [18] propose a method for constructing an FOG temperature drift compensation model based on CEEMDAN, and use an adaptive Kalman filter (AKF) to filter mixed noise, which effectively reduces temperature errors. However, the CEEMDAN has low resolution for high-frequency signals, and the Kalman filter has no ability to improve the resolution of high-frequency signals. Therefore, a CEEMDAN-LWT denoising method combining CEEMDAN and lifting wavelet transform (LWT) is proposed in this study to preprocess the noisy signals of the FOG. The useful IMF components are screened by calculating the correlation coefficient between the IMF components and the original signal. Considering the low resolution of the CEEMDAN method for high-frequency components, the high-frequency IMF components are screened for the lifting wavelet transform to increase the resolution [19]. Then, wavelet soft threshold denoising is performed, and finally, the screened IMF components are reconstructed.

The proposed de-noising method has the complete features of CEEMDAN decomposition. At the same time, the method has the fast de-noising speed and high-resolution features of LWT decomposition. Furthermore, the method achieves the real-time processing of the FOG signal by using a sliding pane. By comparing the results of the denoising effects and Allan variance, the proposed method effectively reduces a majority of the random noise in the FOG signal within the pipe-jacking inertial guidance system. In addition, the proposed method also improves the accuracy of the pipe-jacking attitude results.

## 2. Denoising Principle of CEEMDAN-LWT

### 2.1. CEEMDAN

The CEEMDAN decomposition is achieved in the following steps.

Adding a specific white noise N(t) to the original signal s(t) as the signal to be decomposed, the *i*-th signal can be expressed as follows.

(1) $$s_{i}(t)=s(t)+N_{i}(t)$$

(2) $$N_{i}(t)=\sigma_{k}(t)E_{i}(\varepsilon (t))$$

In Equations (1) and (2), i represents the number of times Gaussian white noise is added, i=1,2…,n; $\sigma_{k}(t)$ is a parameter for adaptive adjustment, k=1,2…,m; ε(t) is the Gaussian white noise; $E_{i}(\varepsilon (t))$ is the *i* th EMD integration average white noise.

First, the first-order IMF component is obtained by EMD decomposition on $s_{i}(t)$, i.e., *IMF_1_*.

(3) $$IMF_{1}=s(t)-\frac{1}{n}\sum_{i=1}^{n}M(s_{i}(t))$$

where *M*(·) represents the local average of the envelope satisfying the *IMF* filtering threshold. The obtained decomposition residue is $r_{1}(t)=s(t)-IMF_{1}$.

Next, the decomposition of the residual $r_{1}(t)$ is continued by adding white noise N(t) and the second-order *IMF* component is obtained.

(4) $$IMF_{2}=r_{1}(t)-\frac{1}{n}\sum_{i=1}^{n}M(r_{1}(t)+N_{i}(t))$$

The above steps are repeated until the residuals cannot be discretized, and the *k*-th order *IMF* components are as follows.

(5) $$IMF_{k}=r_{k}(t)-\frac{1}{n}\sum_{i=1}^{n}M(r_{k}(t)+N_{i}(t))=r_{k-1}(t)-\frac{1}{n}\sum_{i=1}^{n}M(r_{k-1}(t)+\sigma_{k-1}(t)E_{i}(\varepsilon (t)))$$

The *n* is the number of white noises added, which determines the accuracy of the IMF and is usually set between 100 and 1000. Another important factor affecting the decomposition results is $\sigma_{k-1}(t)$. The value of $\sigma_{k-1}(t)$ is usually set between 0.1 and 0.3.

### 2.2. Correlation Analysis of IMF Components

Two signals, *x*(*i*) and *y*(*i*), are set and the correlation coefficient *R* is an indicator of the degree of correlation between the two signals.

(6) $$R=\frac{\sum \left[x(i)-E(x)\right]\left[y(i)-E(y)\right]}{\sqrt{\sum {\left[x(i)-E(x)\right]}^{2}{\left[y(i)-E(y)\right]}^{2}}}$$

When *R* is close to 1, the two signals have a strong positive correlation; when *R* is close to −1, the two signals have a strong negative correlation; and when *R* is closer to 0, the two signals have a nearly weak correlation. After CEEMDAN decomposition, the effective *IMF* components are screened, and pseudo *IMF* components are removed by calculating the correlation coefficient between two signals. This process aims to enhance the accuracy of the signal [20,21].

### 2.3. LWT

The screened *IMF* components are de-noised by lifting wavelet transform, and the lifting algorithm is divided into split, prediction, and updating [22,23].

Step 1: Split. The input signal s(k),k∈Z is divided into two wavelet subsets by parity.

(7) $$\begin{array}{l} s_{o}(k)=s(2k+1),k\in Z\\ s_{e}(k)=s(2k),k\in Z \end{array}$$

where $s_{o}(k)$ is the sequence of odd samples, $s_{e}(k)$ is the sequence of even samples, and *k* is the number of signal samples.

Step 2: Prediction. Set *P* represents the prediction operator, using $s_{e}(k)$ to predict the dual sequence $s_{o}(k)$, and the predicted signal $s_{o}(k)$ is represented as $P[s_{e}(k)]$. The prediction error of the even sample sequence is defined as the detail signal d(k), which is the high-frequency component obtained after transformation.

(8) $$d(k)=s_{o}(k)-P[s_{e}(k)]$$

Step 3: Update. Set *U* represents the update operator that updates d(k) based on the detail signal $s_{e}(k)$ to approximate the overall characteristics of the original signal more closely while maintaining the scale properties of the original signal.

(9) $$c(k)=s_{e}(k)-U[d(k)]$$

where c(k) is the approximation signal and is the low-frequency component obtained from the transform.

### 2.4. Soft Threshold De-Noising and Signal Reconstruction

Soft threshold de-noising is performed on the high-frequency components obtained from the LWT algorithm.

(10) $$\hat{W}=\left\{\begin{array}{c} \mathrm{sgn}(W)(\left|W\right|-\lambda )\\ 0 \end{array}\right.\begin{array}{cc} & \begin{array}{c} \left|W\right|>\lambda \\ \left|W\right|\leq \lambda \end{array} \end{array}$$

where *W* represents the initial wavelet coefficients corresponding to the high-frequency component, $\hat{W}$ denotes the wavelet coefficients of the high-frequency component after threshold processing, and λ represents the threshold.

(11) $$\lambda =\sigma \sqrt{\ln N}$$

where σ is the standardized variance of the noise and N is the signal length.

**Figure 1:**
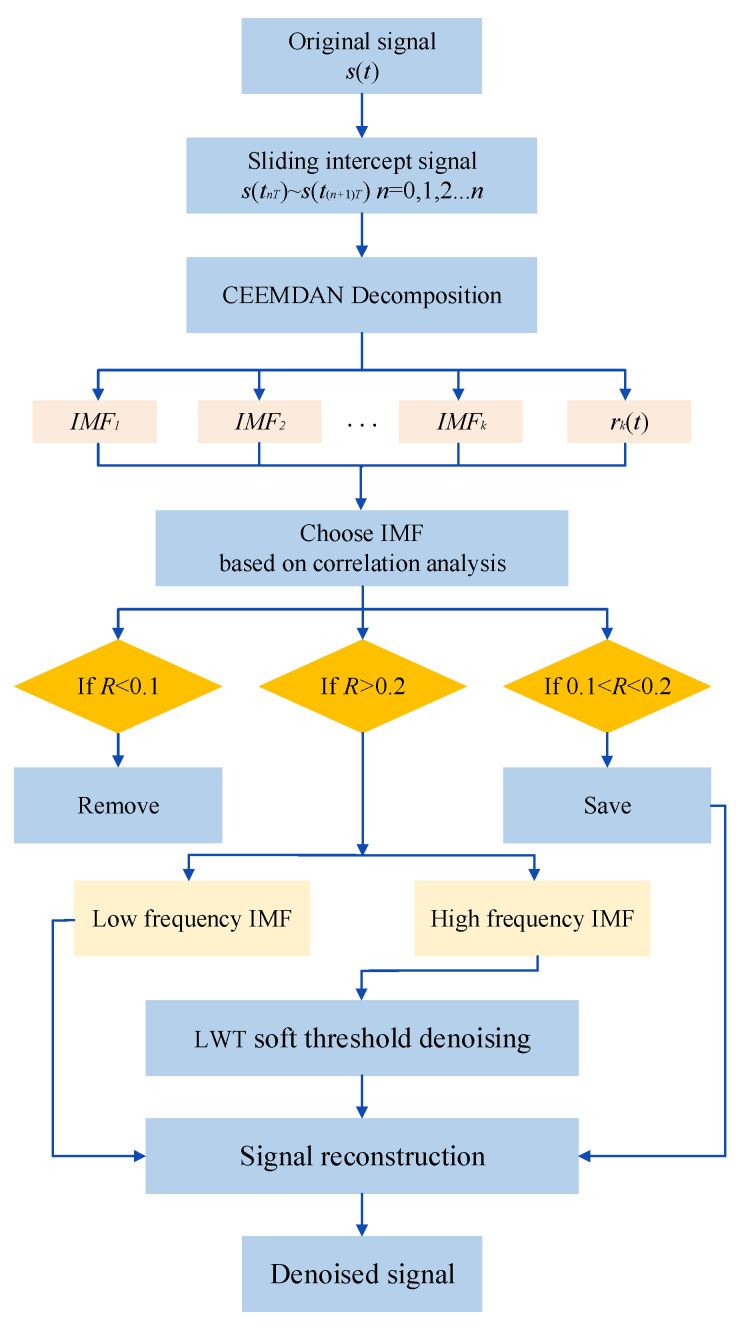
The flowchart of the CEEMDAN-LWT de-noising algorithm.

After soft threshold de-noising, the de-noised signal is obtained by reconstructing each high-frequency component d′(k) and low-frequency component c(k). The flowchart of the CEEMDAN-LWT algorithm is shown in Figure 1.

## 3. Simulation Signal Analysis

**Figure 2:**
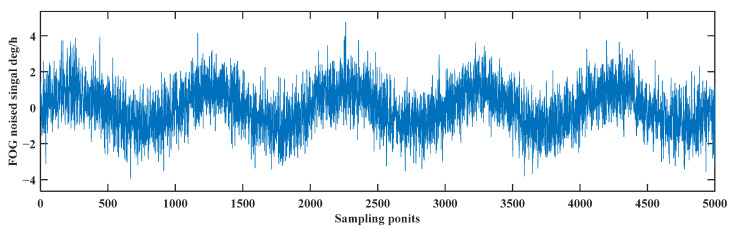
Schematic of the simulated noise signal.

Matlab is used to simulate the FOG signal and verify the effectiveness of the CEEMDAN-LWT de-noising algorithm. The simulated signal is a sinusoidal signal with frequency of 0.1 Hz, amplitude of 1 °/h, sampling frequency of 100 Hz, and sampling time of 50 s. Then, the white noise is added; the mean value of the white noise is 0, and the variance of the white noise is 1 °/h. The noisy signal as shown in Figure 2.

**Figure 3:**
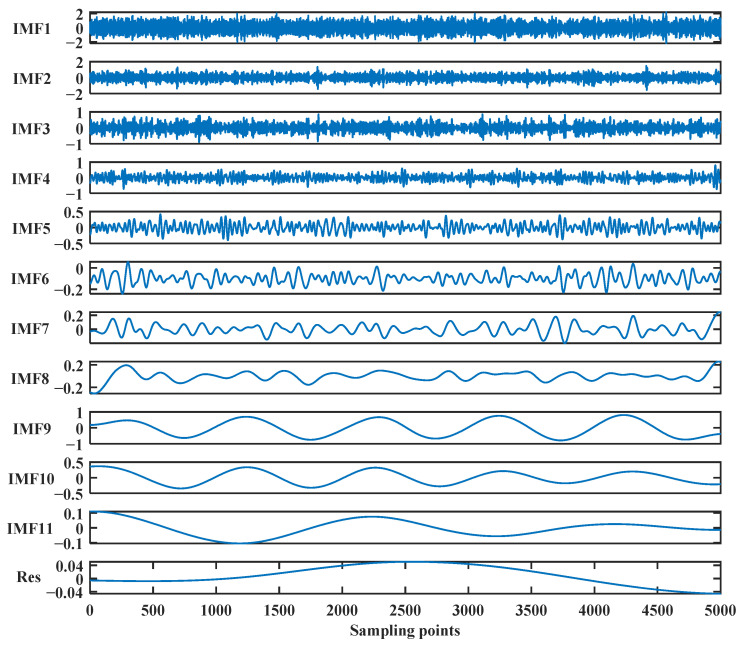
The IMF components and a residue by CEEMDAN decomposition of the simulated signal.

The noisy signal is subsequently conducted to CEEMDAN decomposition, resulting in 11 IMF components and a residue, shown in Figure 3.

In Figure 3, the frequencies of the IMF components gradually decrease from top to bottom, and the last item, Res, is the remaining residuals. It shows that the noise mainly exists in the IMF components at high frequencies, the IMF components at low frequencies contain most of the effective signal, and *IMF*_8_ and *IMF*_9_ are nearly close to the simulated signal. Then, the correlation between each of the obtained IMF components with the noise signal is computed, and the results of the correlation analysis are shown in Table 1.

**Figure 4:**
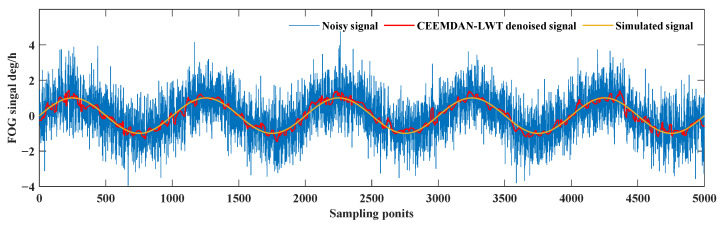
De-noising results of the simulated signal based on the CEEMDAN-LWT method.

According to the results of correlation analysis, the high-frequency *IMF* components: *IMF*_1_, *IMF*_2_, *IMF*_3_, and *IMF*_4_ with a correlation greater than 0.2 are extracted to conduct LWT soft threshold de-noising. The *IMF* components with correlation coefficients less than 0.1 are removed and the other *IMF* components are retained. The signal is reconstructed with the de-noised high-frequency *IMF* components and the remaining *IMF* components. The obtained de-noised signal is compared with the noise signal, and the simulated signal. The compared results are shown in Figure 4.

In Figure 4, the blue curve is the noise-containing signal, the red curve is the signal obtained after de-noising using the CEEMDAN-LWT method, and the yellow curve is the signal without noise in the simulation. The noise of the red curve is significantly reduced compared with the blue curve, and the trend of the red curve is consistent with the yellow curve. The red curve fluctuates around the yellow curve and without signal distortion proves that the algorithm proposed in this study can filter out the noise in the signals well.

**Figure 5:**
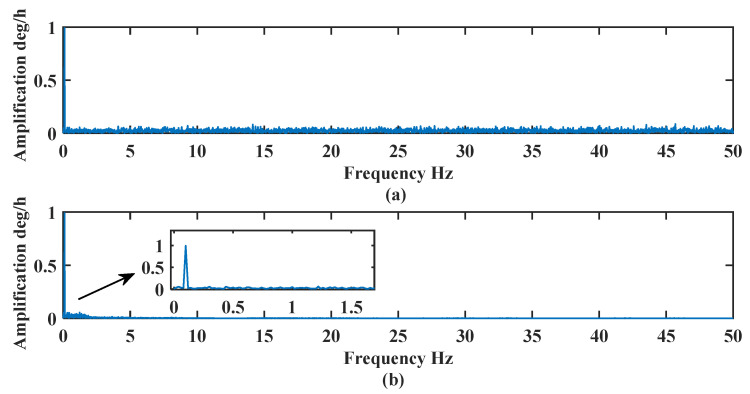
Frequency analysis of noise-containing and de-noised signals: (**a**) noise-containing signal (**b**) denoised signal.

The frequency analysis is conducted on the signals before and after de-noising in Figure 5.

In Figure 5, the frequency of the signal after noise reduction is mainly distributed at 0~0.2 Hz, and the signal frequency set by the simulation signal is 0.1 Hz, so the effective information of the signal is better retained. The signal is better decomposed to different frequencies by the CEEMDAN-LWT and is more effective in removing the high-frequency noise. The signal after noise reduction is close to the real signal, and the features of the effective signal are retained to the maximum extent.

This study applies the signal-to-noise ratio (*SNR*), standard deviation (*SD*), deviation (*D*) and root mean square error (*RMSE*) to identify the effectiveness of the signal denoising. The equations for them are shown in below [24].

(12) $$SNR=10\log_{10}\left(\frac{\sum_{i=1}^{N}s^{2}(i)}{\sum_{i=1}^{N}{\left(s(i)-{s_{t}}^{\prime }(i)\right)}^{2}}\right)$$

(13) $$RMSE=\sqrt{\frac{1}{N}\sum_{i=1}^{N}{\left({s_{t}}^{\prime }(i)-s_{t}(i)\right)}^{2}}$$

(14) $$D=\frac{1}{N}\sum_{i=1}^{N}\left({s_{t}}^{\prime }(i)-{{\hat{s}}_{t}}^{\prime }(i)\right)$$

(15) $$SD=\sqrt{\frac{1}{N}{\sum_{i=1}^{N}\left({s_{t}}^{\prime }(i)-{{\hat{s}}_{t}}^{\prime }(i)\right)}^{2}}$$

where s(i) is the simulated signal, $s_{t}(i)$ is the noise-containing signal, ${s_{t}}^{\prime }(i)$ is the signal after noise reduction, ${{\hat{s}}_{t}}^{\prime }(i)$ is the average value of the signal after noise reduction, and *N* is the number of sampling points. The larger the *SNR*, the smaller the *RMSE*, *SD* and deviation, the better the de-noising effect. The *SNR*, *RMSE*, and deviation represent the de-noising effect of the simulated noise-containing signal. The de-noising effect of the signal is shown in Table 2.

Table 2 compares CEEMDAN, LWT, and CEEMDAN-LWT methods with the de-noising effects. The CEEMDAN method removes the first two high-frequency IMF components directly, and the LWT method conducts a 5-level decomposition and soft thresholds de-noising with the signal. According to *SNR*, *SD*, *RESM*, and *D* results, CEEMDAN-LWT has the best evaluation effect, proving that the algorithm proposed in this study has a better de-noising effect.

## 4. FOG Signal Analysis

### 4.1. Dynamic Pipe-Jacking Experiment 

**Figure 6:**
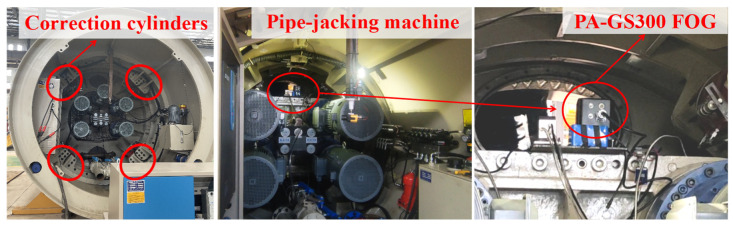
The FOG in pipe-jacking inertial guidance system.

To verify the de-noising effect of the CEEMDAN-LWT method in the pipe-jacking working environment, a PA-GS300 FOG was installed on the running pipe-jacking machine to sample the FOG signal, as shown in Figure 6.

**Figure 7:**
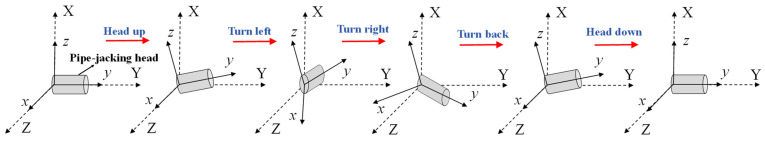
Schematic diagram of pipe-jacking attitude adjustment.

The sampling frequency was 100 Hz. In the dynamic experiment period, the pipe-jacking attitude is adjusted by the correction cylinders. The adjustment steps include setting the pipe-jacking head up first, then adjusting the head to turn from left to right. Finally, the pipe-jacking head is turned back and put down, as shown in Figure 7.

**Figure 8:**
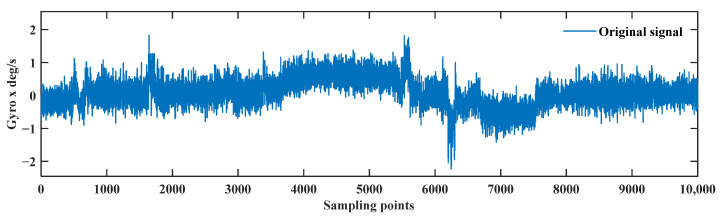
The x-axis gyroscope signal in the dynamic pipe-jacking experiment.

Sliding window data for the CEEMDAN-LWT de-noising method is chosen. The x-axis gyroscope data is taken as an example, as shown in Figure 8.

**Figure 9:**
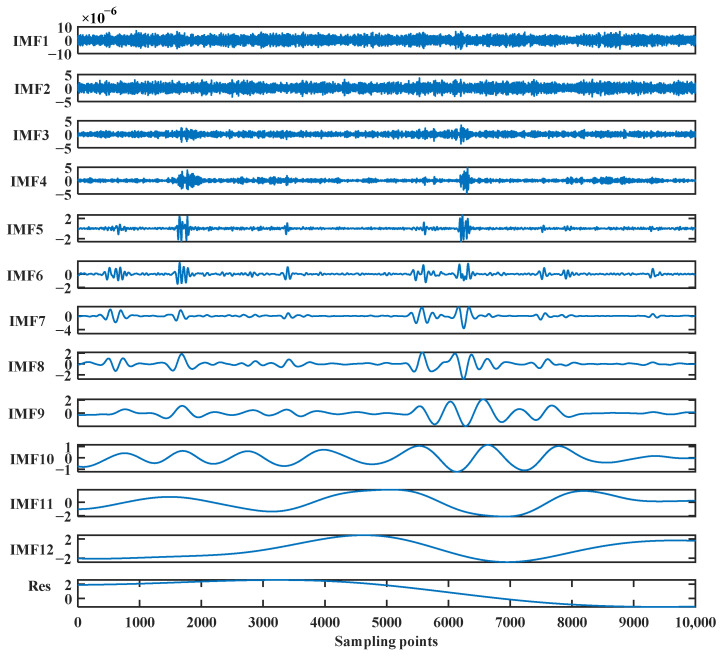
The CEEMDAN decomposed results of the FOG signals for the dynamic pipe-jacking experiment.

The original gyro signal is decomposed using the CEEMDAN method and 12 IMF components and a residue are obtained, as shown in Figure 9.

The correlation analysis of each IMF component with the FOG signal is shown in Table 3.

**Figure 10:**
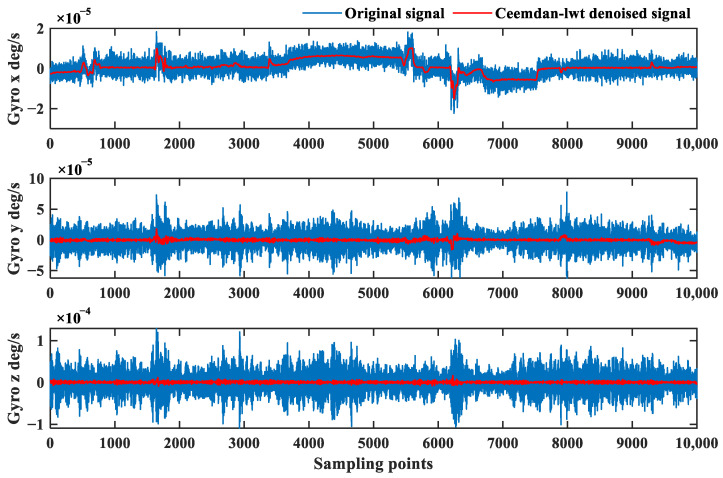
Denoising results of the FOG signal based on the CEEMDAN-LWT method in the dynamic pipe-jacking experiment.

The high-frequency *IMF*_1_, *IMF*_2_, and *IMF*_3_ whose correlation coefficients greater than 0.2 are screened and processed with LWT and soft threshold de-noising. The level of decomposition is 5. The *IMF* components with correlation coefficients of less than 0.1 are removed, and the signal is reconstructed. The de-noised signals are obtained, as shown in Figure 10.

**Figure 11:**
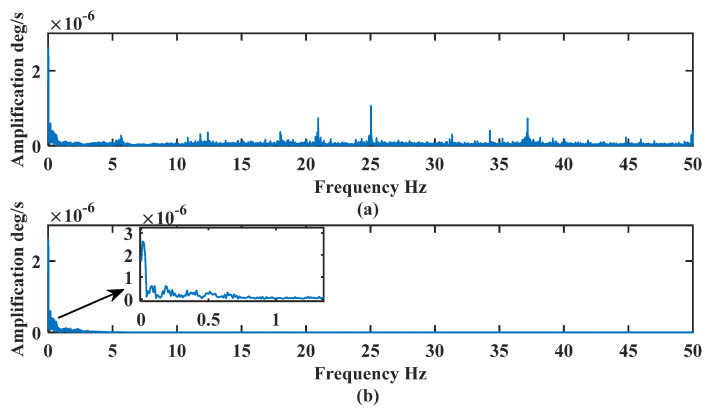
Frequency analysis of the noise-containing and de-noised signals: (**a**) noise-containing signal (**b**) de-noised signal.

Figure 10 shows that the de-noised signal better retains the characteristics of the original signal and filters out most of the noise, which proves that the de-noising method proposed in this paper is suitable for the FOG signals. The frequency analysis of the noise-containing signal and the de-noised signal are shown in Figure 11.

Figure 11a shows that the frequency of the FOG signal is distributed from 0–50 Hz, and the main frequency is distributed near 0 Hz, 21 Hz, 25 Hz, and 37 Hz. During the dynamic experiment, the attitude adjustment is slow, and the effective signal is mainly distributed near 0 Hz. Figure 11b shows that the frequency of the de-noised signal is mainly distributed between 0–1 Hz, and the algorithm filters out most of the high-frequency noise signals, which proves the effectiveness of the algorithm proposed in this paper. The de-noising effect is analyzed and compared in Table 4.

Table 4 compares the de-noising effect of the FOG signal processed using the CEEMDAN, LWT, and CEEMDAN-LWT methods. The CEEMDAN method removes the first two high-frequency *IMF* components of the FOG signal directly, and the LWT method performs a 5-level decomposition and soft thresholding of the FOG signal. According to the SNR and SD results, CEEMDAN-LWT shows the best de-noising effect. It proves that the algorithm proposed in this study effectively improves the de-noising effect. The de-noised signal is solved with inertial navigation to obtain the pipe-jacking attitude, and the results are as follows.

**Figure 12:**
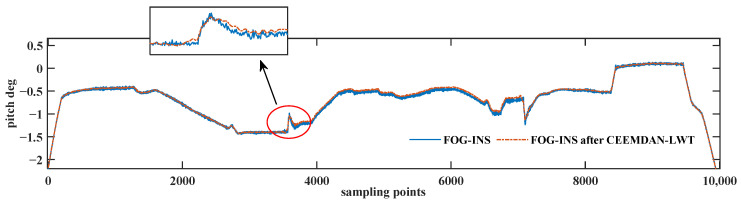
Pitch angle solving results of the dynamic pipe-jacking experiment.

In Figure 12, the blue curve is the result of the inertial navigation solving of the FOG signal, and the red curve is the result of the inertial navigation solving of the FOG signal after de-noising using the CEEMDAN-LWT algorithm. The pitch angle of the FOG signal changes from −2.4° to about −0.5°and then back to −2.4°, which is consistent with the trend of the attitude adjustment, proving that the de-noised signal is not distorted. The detailed graph shows that the fluctuation in the pitch angle solved using the CEEMDAN-LWT algorithm is minor and smoother. It proves that the algorithm proposed in this paper can filter out random noise better.

**Figure 13:**
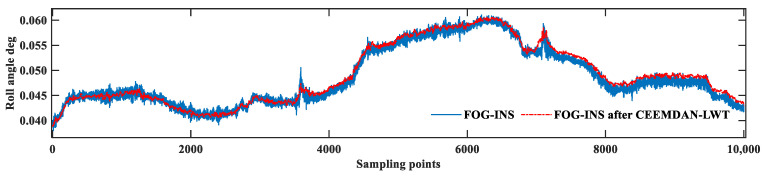
Roll angle solving results of the dynamic pipe-jacking experiment.

In Figure 13, the roll angle is set with no change, and the roll angle after the CEEMDAN-LWT algorithm fluctuates around 0.05°. The trend of the roll angle change before and after de-noising is consistent, which proves that there is no distortion of the FOG signal after de-noising. In the detailed graph, the fluctuation of the red curve is reduced compared with that of the blue curve, which proves that the algorithm proposed in this study is effective.

**Figure 14:**
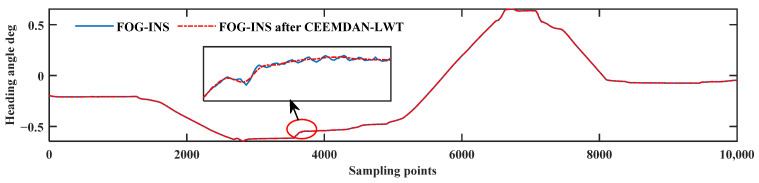
Heading angle solving results of the dynamic pipe-jacking experiment.

In Figure 14, the heading angle changes from −0.2° to −0.7° and then to 0.7° and finally to near 0°, which is consistent with the trend of the attitude adjustment from the left to the right, which proves that there is no distortion after de-noising. In the detailed graph, the heading angle after de-noising is smoother compared with the fluctuation before de-noising, and the algorithm effectively filters out part of the random noise. The attitude de-noising effect of the pipe-jacking dynamic experiment is evaluated as follows.

Table 5 compares the SD of the attitude before and after de-noising; the SD of the attitude after de-noising is reduced, which proves that the proposed algorithm improves the accuracy of attitude results. The SNR results of the de-noised attitude are improved, which proves that the proposed algorithm effectively filters out the random noise.

### 4.2. Pipe-Jacking Environmental Interference Experiment 

**Figure 15:**
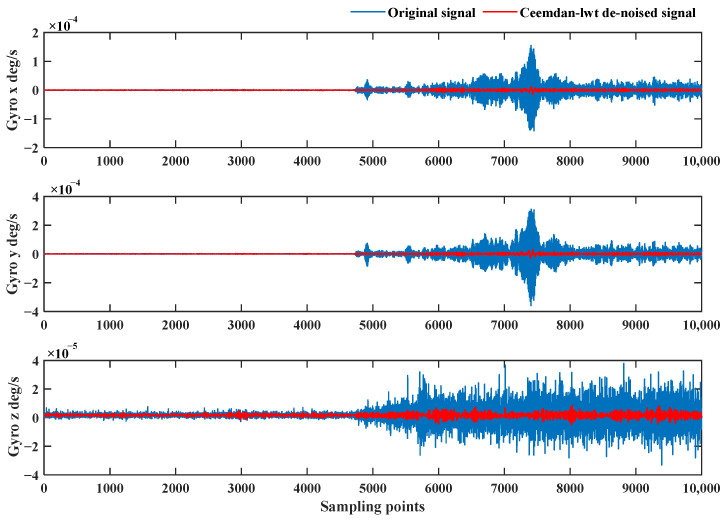
Denoising results of the FOG signal based on the CEEMDAN-LWT method in pipe-jacking environmental interference experiment.

A pipe-jacking environmental interference experiment was conducted to verify the adaptive de-noising effect of the CEEMDAN-LWT algorithm on the FOG during pipe-jacking operations. The sampling frequency was 100 Hz. The 10,000 sample points were intercepted and processed by the algorithm. The frequency converter and the motor were turned on to rotate the pipe jacking cutter at 4650 sampling points. The three-axis data of the FOG after the de-noising by the proposed algorithm are shown in Figure 15.

**Figure 16:**
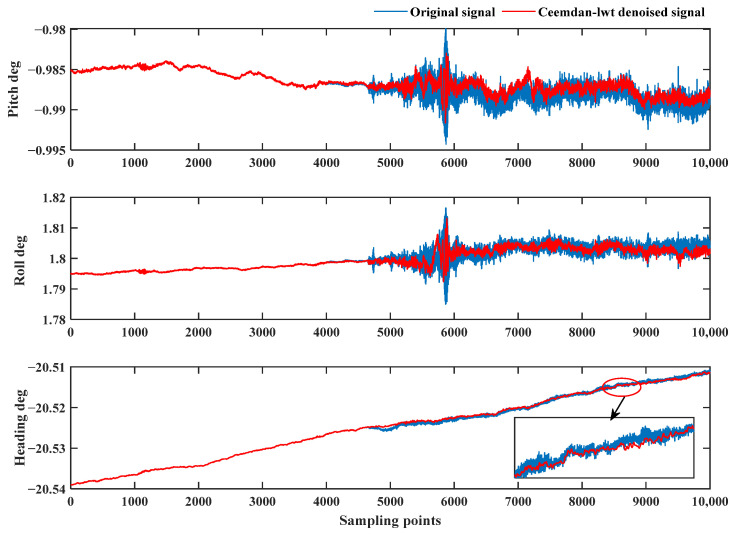
Attitude solving results of the pipe-jacking environmental interference experiment.

The blue curve in Figure 15 is the original signal measured by the FOG, and the noise is enhanced during the pipe-jacking operation at 4650 sampling points. The red curve is the signal after the de-noising algorithm, and the signal amplitude is consistent after de-noising in different environments, proving that the algorithm proposed in this paper has a certain degree of adaptivity. Meanwhile, the proposed algorithm filters out most of the random noise. The attitude results are compared before and after de-noising in Figure 16.

The blue curve in Figure 16 is the attitude result before de-noising. The pipe-jacking operation starts at 4650 sampling points, and the fluctuation of the attitude results is obviously increased. The red curve is the result of attitude solving using the algorithm proposed in this study, and the noise of attitude is obviously reduced, which proves that the de-noising algorithm proposed in this study can effectively filter out the environmental noise. Table 6 is a statistical analysis of the attitude results.

In Table 6, the initial attitude is the result of the attitude at the 4650th sampling point. The ultimate attitude is the result of the attitude at the 10,000th sampling point. The average attitude is the average value of between 4650 and 10,000 sampling points. The pipe-jacking attitude remains static throughout the environmental interference experiment. In theory, the pipe-jacking attitude should not change during the experiment, and the initial attitude value represents the true value. However, due to random noise and gyro drift error, the attitude will drift. By comparing the initial and ultimate attitudes before and after denoising, it is evident that the proposed method reduces a part of the drift error caused by random noise. Table 6 also shows that the average attitude of the CEEMDAN-LWT method is closer to the initial attitude, and the absolute error of the pitch angle is reduced by 39.86% relative to the original signal, and the roll and heading angle are reduced by 59.45% and 14.29%. The maximum relative error of the pitch angle is improved from −0.74% to −0.44%, the roll angle is improved from 2.07% to 0.79%, and the heading angle is improved from −0.07% to −0.06%. It is proven that the CEEMDAN-LWT algorithm proposed in this study filters out the random noise effectively and improves the accuracy of the attitude results. The de-noising effect is evaluated and compared in Table 7.

**Figure 17:**
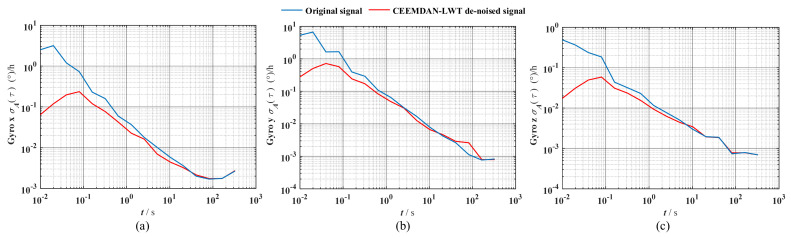
Allan variance curve comparison of the pipe-jacking environmental interference experiment: (**a**) The Allan variance of Gryo X, (**b**) the Allan variance of Gryo Y, (**c**) the Allan variance of Gryo Z.

In Table 7, the CEEMDAN-LWT algorithm has the best de-noising effect compared to the CEEMDAN and LWT algorithms, but the heading angle de-noising effect is weak. Finally, the performance of gyro data before and after de-noising was analyzed using Allan variance as shown in Figure 17.

The blue curve in Figure 17 is the Allan variance curve of the original signal, and the red curve is the Allan variance curve of the CEEMDAN-LWT de-noised signal. Figure 17 shows that the red curves are all in the lower left of the blue curves, which indicates that the noise of the FOG signal de-noised using CEEMDAN-LWT is reduced. Therefore, the proposed de-noising method is effective. The specific error indicators are shown in Table 8.

In Table 8, Q is quantization noise, B is bias instability, N is random walk, and K is the rate of the random walk. The specific noise indicators of the CEEMDAN-LWT denoised signal become smaller, where the value of Q is very small and almost negligible. It is proven that the CEEMDAN-LWT method proposed in this study reduces the noise of the FOG signal effectively.

## 5. Conclusions

According to the characteristics of the FOG signals in the pipe-jacking inertial guidance system, this study proposes a hybrid de-noising method based on CEEMDAN-LWT. The FOG signal is decomposed by CEEMDAN, the main IMF components are screened by correlation coefficients, the high-frequency IMF components in the screening are de-noised using LWT and soft threshold de-noising, and finally, the signal is reconstructed. Simulation, dynamic, and environmental interference experiments were conducted, which compared the analysis of the frequency and the evaluation of the de-noising effect using the CEEMDAN and LWT de-noising methods. The results show that the method proposed in this study can effectively reduce the influence of random noise on the signal and accurately reflect the changing characteristics of the signal. The de-noised pipe-jacking attitude in the environmental interference experiment was closer to the theoretical value, the absolute error of the pitch angle was reduced by 39.86%, the roll angle was reduced by 59.45%, and the heading angle was reduced by 14.29%. The maximum relative error of the pitch angle was improved from −0.74% to −0.44%, the roll angle was improved from 2.07% to 0.79%, and the heading angle was improved from −0.07% to −0.06%. In conclusion, the CEEMDAN-LWT de-noising method proposed in this study combines the CEEMDAN and LWT decomposition features, which have the advantages of complete decomposition, fast speed, and high-resolution. At the same time, the method achieves real-time processing by using a sliding window. The study results show that the proposed method could filter out most of the random noise of the FOG signal in the pipe-jacking inertial guidance system and improve the accuracy of the pipe-jacking attitude results, which has better engineering application value.

## Figures and Tables

**Table 1:** Results of correlation analysis.

*IMF* Number	Correlation	*IMF* Number	Correlation
*IMF*_1_	0.64	*IMF*_7_	0.13
*IMF*_2_	0.47	*IMF*_8_	0.22
*IMF*_3_	0.35	*IMF*_9_	0.57
*IMF*_4_	0.27	*IMF*_10_	0.54
*IMF*_5_	0.19	*IMF*_11_	0.10
*IMF*_6_	0.14	Res	0.06

**Table 2:** Compared results of denoising effect evaluation.

Method	*SNR* (dB)	*RMSE* (deg/h)	*D* (deg/h)	*SD* (deg/h)
CEEMDAN	8.52	0.287	0.012	0.85
LWT	7.68	0.311	0.016	0.87
CEEDAN-LWT	10.55	0.210	0.006	0.75

**Table 3:** Correlation analysis results of each IMF component.

*IMF* Number	Correlation	*IMF* Number	Correlation
*IMF*_1_	0.53	*IMF*_8_	0.24
*IMF*_2_	0.43	*IMF*_9_	0.26
*IMF*_3_	0.26	*IMF*_10_	0.19
*IMF*_4_	0.18	*IMF*_11_	0.50
*IMF*_5_	0.13	*IMF*_12_	0.61
*IMF*_6_	0.15	Res	0.36
*IMF*_7_	0.21		

**Table 4:** Comparison of denoising effect in the dynamic pipe-jacking experiment.

Method	*SNR* (dB)	*SD* (deg/s)
CEEMDAN	13.01	1.28 × 10^−6^
LWT	11.94	1.30 × 10^−6^
CEEDAN-LWT	18.18	1.19 × 10^−6^

**Table 5:** Evaluation of the attitude denoising effect in the dynamic pipe-jacking experiment.

	Attitude	*SD* (deg/s)	*SNR* (dB)
Original signal	Pitch	0.4469	-
	Roll	0.0077	-
	Heading	0.0036	-
Denoised signal	Pitch	0.4461	28.3121
	Roll	0.0076	33.5005
	Heading	0.0035	10.0399

**Table 6:** Statistics of attitude results in the pipe-jacking environmental interference experiment.

Signal	Attitude Angle	Initial Attitude/deg	Ultimate Attitude/deg	Average Attitude/deg	Absolute Error/deg	Relative Error/%
Original signal	Pitch	−0.9873	−0.9896	−0.9880	−0.0070~0.0073	−0.74~0.71
	Roll	1.7992	1.8057	1.8024	−0.0140~0.0373	−0.78~2.07
	Heading	−20.5249	−20.5111	−20.5188	0~0.0138	−0.07~0
CEEMDAN-LWT denoised signal	Pitch	−0.9873	−0.9874	−0.9875	−0.0043~0.0043	−0.44~0.44
	Roll	1.7992	1.8005	1.8021	−0.0068~0.0140	−0.38~0.79
	Heading	−20.5249	−20.5118	−20.5186	0~0.0131	−0.06~0

**Table 7:** Evaluation of the attitude denoising effect in the pipe-jacking environmental interference experiment.

Method	Attitude	*SNR* (dB)	*SD* (deg)	*D* (deg)
CEEMDAN	Pitch	13.4520	0.0010	−0.0008
	Roll	13.1435	0.0023	0.0024
	Heading	21.6513	0.0044	0.0063
LWT	Pitch	13.3676	0.0011	−0.0009
	Roll	12.6715	0.0025	0.0029
	Heading	21.7531	0.0045	0.0065
CEEDAN-LWT	Pitch	13.5926	0.0009	−0.0002
	Roll	13.2295	0.0018	0.0012
	Heading	22.3470	0.0042	0.0063

**Table 8:** Comparison of Allan variance between the original signal and CEEMDAN-LWT denoised signal.

	**Original Signal**			**CEEMDAN-LWT Denoised Signal**		
Gyro axial	Gyro x	Gyro y	Gyro z	Gyro x	Gyro y	Gyro z
Q/(″)	0.003	0.031	0.003	-	-	-
N/deg/h^1/2^	3.436 × 10^−3^	7.809 × 10^−3^	8.740 × 10^−4^	1.118 × 10^−3^	2.703 × 10^−3^	2.760 × 10^−4^
B/deg/h	1.762 × 10^−2^	7.964 × 10^−4^	7.382 × 10^−4^	1.694 × 10^−2^	5.446 × 10^−4^	6.182 × 10^−4^
K/deg/h^3/2^	8.359 × 10^−2^	9.730 × 10^−2^	4.484 × 10^−2^	7.461 × 10^−2^	8.655 × 10^−2^	4.441 × 10^−2^

## Data Availability

Data are contained within the article.
